# Editorial: Past, present, and future of Brugada syndrome: a comprehensive framework

**DOI:** 10.3389/fcvm.2026.1851845

**Published:** 2026-06-29

**Authors:** Vincenzo Santinelli, Mélèze Hocini

**Affiliations:** 1 Arrhythmology Department, IRCCS Policlinico San Donato, Milan, Italy; 2 Clinical Arrhythmia Department, University Hospital Bordeaux, Bordeaux, France

**Keywords:** Brugada syndrome, cardiac arrest, sudden cardiac death, syncope, ventricular fibrillation

For decades, the surface electrocardiogram has served as a window into the mechanisms of sudden cardiac death, particularly in young individuals with structurally normal hearts. From the identification of accessory pathways in Wolff–Parkinson–White syndrome to the recognition of arrhythmic risk in Long QT Syndrome, Early Repolarization syndrome, and Brugada syndrome, a single electrical signal has often provided the first clue to the presence of a life-threatening arrhythmogenic substrate. The challenge has never been merely recognizing the pattern, but understanding the mechanism behind it.

Brugada syndrome represents one of the most intriguing examples of this principle. For many years the disease appeared to be defined almost exclusively by its typical electrocardiographic phenotype. Only recently has the underlying arrhythmogenic substrate begun to emerge, reshaping our understanding of both the disease mechanism and its clinical implications.

## 1 Past: historical foundations and early recognition

### 1.1 Electrocardiographic discovery and diagnostic challenges

The historical narrative of Brugada syndrome (BrS) in many respects parallels that of Wolff–Parkinson–White (WPW) syndrome. In WPW, the arrhythmogenic substrate—the accessory pathway—was anatomically identifiable. Early studies in asymptomatic individuals demonstrated that careful electrophysiological characterization of pathway properties could independently predict the risk of sudden cardiac death even in the absence of symptoms (1–6). These findings influenced international guidelines and established a fundamental principle of clinical electrophysiology: a single electrical signal can reveal the mechanisms underlying life-threatening arrhythmias (1).

It was during my training under the mentorship of John J. Gallagher and Fred Morady that I first learned to look beyond the surface electrocardiogram. They emphasized that subtle electrical signals often contain the roadmap to ventricular fibrillation (VF) and sudden cardiac death. This perspective later using high-density three-dimensional mapping guided investigations worldwide in understanding diagnosis, prognosis and therapy of BrS (7–11).

For many years, however, BrS appeared fundamentally different from WPW. While WPW was defined by a clearly identifiable anatomical pathway on surface ECG, BrS seemed to be characterized solely by its electrocardiographic phenotype.

Early observations described the type-1 ECG pattern primarily in patients resuscitated from VF or in small cohorts of predominantly symptomatic patients with implantable cardioverter-defibrillators. The spontaneous type-1 ECG pattern became both the diagnostic hallmark of the disease and the principal marker of arrhythmic risk. Yet the absence of a clearly defined substrate made mechanistic interpretation difficult. However, the prevalence and prognostic significance of this finding in asymptomatic individuals remained uncertain. Without knowledge of the underlying substrate, it was impossible to determine whether the ECG pattern represented a transient electrical phenomenon or the manifestation of a fixed arrhythmogenic substrate capable of sustaining ventricular fibrillation.

### 1.2 Early risk stratification paradigm

As a consequence, risk stratification relied largely on clinical features and probabilistic combinations of variables, often leading to uncertainty and inconsistent recommendations. This binary approach, viewing patients as either “symptomatic” (and therefore high risk) or “asymptomatic” (and therefore low-risk) dominated early clinical practice, but it became increasingly clear that this oversimplification failed to capture the heterogeneous nature of disease expression.

## 2 Present: current understanding and contemporary approaches

### 2.1 Substrate recognition and mechanistic paradigm shift

Over the past decade, this conceptual framework has begun to change dramatically (7–12). Advances in high-density epicardial mapping have revealed the presence of abnormal electrograms localized to the right ventricular outflow tract. These signals are characterized by low voltage, fractionation, and delayed activation, reflecting areas of slow and heterogeneous conduction capable of sustaining life-threatening ventricular arrhythmias and VF. Recognition of this arrhythmogenic substrate has profoundly reshaped our understanding of BrS. The disease is no longer viewed solely as an electrocardiographic phenotype but increasingly as a substrate-dependent arrhythmogenic disorder.

### 2.2 Therapeutic advances: epicardial substrate ablation

Several observational and randomized studies involving large cohorts of highly symptomatic patients have demonstrated remarkable success with epicardial substrate ablation (10–12). In many cases, elimination of the abnormal substrate results not only in suppression of recurrent ventricular arrhythmias but also in normalization of the ECG pattern. These findings strongly support a causal relationship between substrate extent and the electrocardiographic phenotype, suggesting that the type-1 ECG pattern may represent the surface manifestation of the underlying arrhythmogenic substrate rather than simply a diagnostic marker.

### 2.3 Evolution of genetic understanding

Nevertheless, important challenges remain, particularly in asymptomatic individuals. Recent guideline revisions have attempted to incorporate some of these insights by refining diagnostic criteria and reducing the diagnostic weight of drug-induced ECG patterns in the absence of clinical manifestations (7). However, reliable strategies for risk stratification in asymptomatic patients remain limited. The contributions presented in this thematic series highlight the complexity of the field. Several reviews emphasize persistent diagnostic challenges, including the limited specificity of the ECG phenotype and the modest yield of genetic testing, with pathogenic variants in SCN5A identified in only a minority of patients.

### 2.4 Contemporay risk stratification models

Other articles explore evolving strategies for risk stratification that integrate clinical, electrocardiographic, electrophysiological, and genetic variables. Yet the predictive performance of these models remains inconsistent.

- Shangai score: Established in 2017 to address concerns about overdiagnosis, this point-based system incorporates clinical features and genetic data with higher cumulative scores indicating probable or definite BrS.
- Sieira score: derived from a large Spanish registry of nearly 1,000 patients, it incorporates six variables (spontaneous type-1 ECG, syncope, sinus node dysfunction, aborted SCA, early familial history of SCD, and EPS inducibility with a C-statistic of approximatively 0.73).
- Brugada risk score: Developed form a multinational cohort exceeding 2,000 patients using advanced statistical and machine learning methods, it demonstrated C statistics of 0.81.
- PAT (Predicting arrhythmic event) score: Derived from a worldwide pooled analysis of 7,358 patients, a PAT score ≥10 predicted the first major arrhythmic event with 95.5% sensitivity and 89.1% specificity.

### 2.5 Diagnostic complexity and temporal dynamics

An additional layer of complexity arises from the temporal variability of the ECG pattern. In many patients, the type-1 ECG fluctuates in response to fever, medications, metabolic disturbances, or autonomic influences. In others, the pattern remains persistently present. This distinction may be clinically meaningful as persistent ECG expression likely reflects a fixed and extensive arrhythmogenic substrate, whereas fluctuating patterns may indicate a more dynamic electrophysiological state.

Understanding these temporal dynamics may therefore represent a crucial step toward distinguishing patients at true risk of malignant arrhythmias from those in whom the ECG pattern reflects a transient or less dangerous condition.

## 3 Future: emerging directions and personalized management

### 3.1 The asymptomatic patient paradox

The management of asymptomatic individuals remains one of the most challenging aspects of BrS. Many patients are diagnosed incidentally during family screening or routine clinical evaluation, yet reliable markers for predicting malignant arrhythmic events remain limited.

This discrepancy between the strong association of the type-1 ECG pattern with VF and the relatively low incidence of events among asymptomatic individuals represents a persistent clinical paradox.

### 3.2 Mechanistic integration and substrate characterization

The historical experience with WPW syndrome provides a useful analogy. In WPW, identification and characterization of the accessory pathway ultimately transformed both risk stratification and therapeutic strategies. A similar mechanistic approach is now emerging in BrS, integrating careful analysis of the ECG with characterization of the underlying arrhythmogenic substrate. Technological advances may further accelerate this transition.

### 3.3 Advanced technological approaches

Electro-anatomical mapping, advanced imaging, and computational ECG analysis offer the possibility of linking surface electrical patterns with structural and functional properties of the substrate. These approaches move beyond descriptive ECG criteria toward a more mechanistic understanding of the disease.

Large international registries integrating clinical, genetic, and electrophysiological data will be essential to validate emerging predictors and guide future therapeutic strategies.

### 3.4 Genetic and genomic evaluation

Future management strategies are likely to be increasing personalized. Mutation-specific analyses within SCN5A and polygenic scores hold promise for more individualized risk assessment. Emerging research should focus on exploring ways to directly target genetic defects. Using advanced genetic tools such as CRISPR, delivered through heart-targeted viral vectors may offer a transition from symptomatic management toward potentially curative strategies.

### 3.5 Artificial intelligence and precision diagnostics

Artificial intelligence applied to ECG interpretation is another promising avenue. Early studies show how AI can reliably detect BrS patterns raising the possibility of non-invasive screening and early risk identification. Combining AI with polygenic risk scores could refine risk prediction especially for asymptomatic or intermediate-risk patients. At a population level, large genomic datasets combined with AI and polygenic modeling could support predictive testing for SCD-associated SCN5A variants enabling preventive strategies before symptoms appear. Looking toward the future, the most promising advances will likely come from combining dynamic physiologic data streams, interpretable AI models, imaging biomarkers and genomic context, all trained and validated in large, diverse datasets.

### 3.6 Alternative therapeutic strategies

Finally, procedural approaches such as right ventricular outflow tract epicardial ablation may provide alternatives to ICD implantation or reduction of ICD shocks in select high-risk patients. Several observational and randomized studies involving large cohorts of highly symptomatic patients have demonstrated remarkable success with epicardial substrate ablation with elimination of the abnormal substrate resulting not only in suppression of recurrent ventricular arrhythmias but also in normalization of the electrocardiographic pattern (10–12). Current guidelines recommend catheter ablation of the RVOT epicardial substrate in BrS patients with recurrent appropriate ICD shocks refractory to drug therapy as a Class IIa recommendation.

### 3.7 Investigational approaches

Another area of ongoing investigation concerns the potential role of autoantibodies in BrS (13). Although mechanistically intriguing, this field remains largely experimental. Current clinical data do not demonstrate a clear relationship between autoantibody presence and the ECG phenotype. Importantly, these biomarkers do not capture the temporal stability or variability of the type-1 ECG pattern—features that may represent critical determinants of arrhythmic risk. Premature reliance on such markers could therefore increase false-positive diagnoses without improving risk prediction.

## 4 Conclusion: from uncertainty to mechanism, from mechanism to prevention

Brugada Syndrome stands at a critical juncture. The field has transitioned from viewing the disease as purely electrocardiographic to recognizing an underlying substrate-dependent arrhythmogenic disorder. Yet substantial gaps persist, particularly in our ability to distinguish between truly at risk asymptomatic patients and those destined to remain event-free. The emerging convergence of mechanistic understanding, improved risk stratification models, advanced therapeutic options and technological innovation offers promise for more precise and personalizes patient management. However, this progress must remain grounded in rigorous validation, appropriate caution regarding model extrapolation and careful attention to patient values and preferences. As the surface 12-lead ECG continues to guide our understanding of ventricular arrhythmias, it is the integration of this electrical signal with knowledge of the underlying substrate, both electrical and genetic, that will ultimately transform uncertainty into mechanism and mechanism into prevention.

## References

[1] Santinelli V. European Perspective in cardiology: Vincenzo Santinelli, MD. Pioneer in clinical cardiac electrophysiology. Circulation. (2012) 125(18):F103–6.

[2] Pappone C Santinelli V Rosanio S Vicedomini G Nardi S Pappone A. Usefulness of invasive electrophysiologic testing to stratify the risk of arrhythmic events in asymptomatic patients with Wolff-Parkinson-white pattern: results from a large prospective long-term follow-up study. J Am Coll Cardiol. (2003) 41(2):239–44. 10.1016/s0735-1097(02)02706-7 12535816

[3] Pappone C Santinelli V Manguso F Augello G Santinelli O Vicedomini G. A randomized study of prophylactic catheter ablation in asymptomatic patients with the Wolff-Parkinson-white syndrome. N Engl J Med. (2003) 349(19):1803–11. 10.1056/NEJMoa035345 14602878

[4] Pappone C Manguso F Santinelli R Vicedomini G Sala S Paglino G. Radiofrequency ablation in children with asymptomatic Wolff-Parkinson-white syndrome. N Engl J Med. (2004) 351(12):1197–205. 10.1056/NEJMoa040625 15371577

[5] Pappone C Santinelli V. Should catheter ablation be performed in asymptomatic patients with Wolff-Parkinson-white syndrome? Catheter ablation should be performed in asymptomatic patients with Wolff-Parkinson-white syndrome. Circulation. (2005) 112(14):2207–15; discussion 2216. 16208793

[6] Santinelli V Radinovic A Manguso F Vicedomini G Gulletta S Paglino G. The natural history of asymptomatic ventricular pre-excitation a long-term prospective follow-up study of 184 asymptomatic children. J Am Coll Cardiol. (2009) 53(3):275–80. 10.1016/j.jacc.2008.09.037 19147045

[7] Zeppenfeld K Tfelt-Hansen J de Riva M Winkel BG Behr ER Blom NA. ESC 2022 guidelines for the management of patients with ventricular arrhythmias and the prevention of sudden cardiac death. Eur Heart J. (2022) 43:3997–4126. 10.1093/eurheartj/ehac262 36017572

[8] Pappone C Brugada J Vicedomini G Ciconte G Manguso F Saviano M. Electrical substrate elimination in 135 consecutive patients with Brugada syndrome. Circ Arrhythm Electrophysiol. (2017) 10:e005053. 10.1161/CIRCEP.117.005053 28500178

[9] Pappone C Ciconte G Manguso F Vicedomini G Mecarocci V Conti M. Assessing the malignant arrhythmic substrate in patients with Brugada syndrome. J Am Coll Cardiol. (2018) 71:1631–46. 10.1016/J.Jacc.2018.02.022 29650119

[10] Santinelli V Ciconte G Manguso F Anastasia L Micaglio E Calovic Z. High-risk Brugada syndrome: factors associated with arrhythmia recurrence and benefits of epicardial ablation in addition to ICD implantation. Europace. (2024) 26. 10.1093/europace/euae019 PMC10824473 38252933

[11] Nademanee K Chung FP Sacher F Nogami A Nakagawa H Jiang C. Long-term outcomes of Brugada substrate ablation: a report from BRAVO (Brugada ablation of VF substrate ongoing multicenter registry). Circulation. (2023) 147:1568–78. 10.1161/CIRCULATIONAHA.122.063367 36960730

[12] Nademanee K Wongcharoen W Chimparlee N Chokesuwattanaskul R Annueypol M Phusunti K. Brugada syndrome ablation for the prevention of ventricular fibrillation episodes (BRAVE). Heart Rhythm. (2025) 22(8):1975–83. 10.1016/j.hrthm.2025.04.033 40294736

[13] Tarantino A Ciconte G Melgari D Frosio A Ghiroldi A Piccoli M. Nav1.5 autoantibodies in Brugada syndrome: pathogenetic implications. Eur Heart J. (2024) 45(40):4336–48. 10.1093/eurheartj/ehae480 39078224 PMC11491155

